# 24-month intervention with a specific multinutrient in people with prodromal Alzheimer's disease (LipiDiDiet): a randomised, double-blind, controlled trial

**DOI:** 10.1016/S1474-4422(17)30332-0

**Published:** 2017-12

**Authors:** Hilkka Soininen, Alina Solomon, Pieter Jelle Visser, Suzanne B Hendrix, Kaj Blennow, Miia Kivipelto, Tobias Hartmann, LipiDiDiet clinical study group, Ilona Hallikainen, Merja Hallikainen, Seppo Helisalmi, Tarja Lappalainen, Yawu Liu, Teemu Paajanen, Lars-Olof Wahlund, Yvonne Freund-Levi, Niels Andreasen, Göran Hagman, Stina Lindblom, Klaus Fassbender, Matthias Riemenschneider, Marcus OW Grimm, Aline Klees-Rollmann, Maxine Luley, Epameinondas Lyros, Robert Schomburg, Jennifer Kennel, Daniela Ramelli, Lutz Frölich, Lucrezia Hausner, Christoph Laske, Thomas Leyhe, Christian Mychajliw, Niklas Koehler, Stephan Schiekofer, Hans Klünemann, Johannes Schröder, Dieter Lütjohann, Philip Scheltens, Ineke van Rossum, Nienke Scheltens, Daniela Bertens, Mara ten Kate, Frederik Barkhof, Johanna ML Henselmans, Gerwin Roks, Anneke MJ van Hees, Noel Ellison

**Affiliations:** a Department of Neurology, Institute of Clinical Medicine, University of Eastern Finland, Kuopio, Finland; b Neurocenter, Department of Neurology, Kuopio University Hospital, Kuopio, Finland; c Department of Clinical Geriatrics, Department of Neurobiology, Care Sciences and Society, Karolinska Institute, Huddinge, Sweden; d Clinical Trials Unit, Department of Geriatric Medicine, Karolinska University Hospital, Huddinge, Sweden; e Department of Psychiatry and Neuropsychology, Alzheimer Center Limburg, University of Maastricht, Maastricht, Netherlands; f Department of Neurology, Alzheimer Center, Amsterdam Neuroscience, VU University Medical Center, Amsterdam, Netherlands; g Pentara Corporation, Salt Lake City, UT, USA; h Department of Psychiatry and Neurochemistry, Institute of Neuroscience and Physiology, The Sahlgrenska Academy at University of Gothenburg, Mölndal, Sweden; i Clinical Neurochemistry Laboratory, Sahlgrenska University Hospital, Mölndal, Sweden; j German Institute for Dementia Prevention (DIDP), Medical Faculty, and Department of Experimental Neurology, Saarland University, Homburg, Germany

## Abstract

**Background:**

Nutrition is an important modifiable risk factor in Alzheimer's disease. Previous trials of the multinutrient Fortasyn Connect showed benefits in mild Alzheimer's disease dementia. LipiDiDiet investigated the effects of Fortasyn Connect on cognition and related measures in prodromal Alzheimer's disease. Here, we report the 24-month results of the trial.

**Methods:**

LipiDiDiet was a 24-month randomised, controlled, double-blind, parallel-group, multicentre trial (11 sites in Finland, Germany, the Netherlands, and Sweden), with optional 12-month double-blind extensions. The trial enrolled individuals with prodromal Alzheimer's disease, defined according to the International Working Group (IWG)-1 criteria. Participants were randomly assigned (1:1) to active product (125 mL once-a-day drink containing Fortasyn Connect) or control product. Randomisation was computer-generated centrally in blocks of four, stratified by site. All study personnel and participants were masked to treatment assignment. The primary endpoint was change in a neuropsychological test battery (NTB) score. Analysis was by modified intention to treat. Safety analyses included all participants who consumed at least one study product dose. This trial is registered with the Dutch Trial Register, number NTR1705.

**Findings:**

Between April 20, 2009, and July 3, 2013, 311 of 382 participants screened were randomly assigned to the active group (n=153) or control group (n=158). Mean change in NTB primary endpoint was −0·028 (SD 0·453) in the active group and −0·108 (0·528) in the control group; estimated mean treatment difference was 0·098 (95% CI −0·041 to 0·237; p=0·166). The decline in the control group was less than the prestudy estimate of −0·4 during 24 months. 66 (21%) participants dropped out of the study. Serious adverse events occurred in 34 (22%) participants in the active group and 30 (19%) in control group (p=0·487), none of which were regarded as related to the study intervention.

**Interpretation:**

The intervention had no significant effect on the NTB primary endpoint over 2 years in prodromal Alzheimer's disease. However, cognitive decline in this population was much lower than expected, rendering the primary endpoint inadequately powered. Group differences on secondary endpoints of disease progression measuring cognition and function and hippocampal atrophy were observed. Further study of nutritional approaches with larger sample sizes, longer duration, or a primary endpoint more sensitive in this pre-dementia population, is needed.

**Funding:**

European Commission 7th Framework Programme.

## Introduction

The course of Alzheimer's disease is characterised by progressive accumulation of neuropathology over decades. The initial asymptomatic phase continues into a prodromal phase with mild, but noticeable, cognitive and functional impairment,^1^ and eventually progression to dementia. This gradual progression creates a window of opportunity for interventions in early disease stages.^2^ Specific criteria to define the prodromal phase of Alzheimer's disease have been proposed using biomarkers and clinical criteria,^3^, ^4^, ^5^ but no pharmacological treatment is currently available for individuals with prodromal Alzheimer's disease. Development of safe and effective interventions in early Alzheimer's disease stages remains imperative. Prevention trials from the past 2 years have shown promising results with multimodal, non-pharmacological approaches, including dietary interventions.^6^, ^7^

Diet is an important modifiable risk factor for dementia,^8^ and a nutrient intervention in mild cognitive impairment showed effects on brain atrophy.^9^ LipiDiDiet is a research consortium, which studies the preclinical and clinical impact of nutrition in Alzheimer's disease. This research resulted in experimental dietary interventions, which contributed to the development of the medical food Souvenaid (Nutricia; Zoetermeer, the Netherlands). The active component of Souvenaid is the multinutrient combination (Fortasyn Connect), which contains docosahexaenoic acid (DHA); eicosapentaenoic acid (EPA); uridine monophosphate; choline; vitamins B12, B6, C, E, and folic acid; phospholipids; and selenium.^10^ These nutrients were selected based on their established biological and neuroprotective properties, and specifically combined to enhance efficacy compared with individual nutrients. The aim was to provide neuroprotection by targeting disease processes in early Alzheimer's disease—ie, by supplying rate-limiting compounds for brain phospholipid synthesis and addressing multiple Alzheimer's disease-related pathological processes in vivo.^11^, ^12^, ^13^, ^14^, ^15^, ^16^, ^17^ Results from animal studies showed that this multinutrient combination improved neuronal membrane composition; increased the formation of synapses, cholinergic neurotransmission, and cerebral blood flow and perfusion; preserved neuronal integrity; restored hippocampal neurogenesis; reduced β-amyloid pathology; and improved cognition.^15^, ^16^, ^17^, ^18^, ^19^, ^20^, ^21^ Concentrations of these nutrients in plasma and CSF or the brain were also found to be lower in patients with Alzheimer's disease.^22^ For clinical use, Fortasyn Connect was adapted to address nutritional requirements in the presence of Alzheimer's disease pathology. Two previous randomised clinical trials in patients with mild Alzheimer's disease dementia reported that daily intake of Fortasyn Connect for 3 or 6 months improved memory performance,^23^, ^24^ increased neurophysiological measures of synaptic activity, and enhanced functional connectivity in the brain.^24^, ^25^ A third randomised controlled trial^26^ in patients with mild-to-moderate Alzheimer's disease dementia did not report benefits, therefore, heterogeneity in the benefits of Fortasyn Connect exists in previous trials. All trials reported a positive safety profile^23^, ^24^, ^27^ and treatment was well tolerated in combination with Alzheimer's disease medications.^26^ An analysis of these trials indicated that Fortasyn Connect can achieve clinically detectable effects in patients with mild Alzheimer's disease dementia,^28^ but did not slow cognitive decline in mild-to-moderate Alzheimer's disease dementia.^26^ Given the hypothesis that earlier intervention might be more beneficial, the LipiDiDiet trial was designed to investigate the effects of Fortasyn Connect on cognition and related measures in prodromal Alzheimer's disease.

**Research in context**

**Evidence before this study**

We searched ClinicalTrials.gov, WHO's International Clinical Trial Registry Platform, and PubMed (Jan 1, 1950, to Dec 20, 2016) using the search terms “Alzheimer's disease” and “Fortasyn” or “Souvenaid”. There were no language restrictions. Only articles reporting clinical trials of Souvenaid or Fortasyn Connect in patients with Alzheimer's disease were included. We identified three completed 12-week to 24-week randomised controlled trials: Souvenir I (225 drug-naive patients with mild Alzheimer's disease dementia), Souvenir II (259 drug-naive patients with mild Alzheimer's disease dementia), and S-Connect (527 patients with mild-to-moderate Alzheimer's disease dementia treated with medications). Improved memory was reported in mild, but not mild-to-moderate, Alzheimer's disease. The three randomised controlled trials reported that the intervention was well tolerated and had a good safety profile, both alone and in combination with Alzheimer's disease medications. The LipiDiDiet trial differs from the previous trials of this multinutrient combination because it focuses on prodromal Alzheimer's disease and tests a longer duration of the intervention.

**Added value of this study**

LipiDiDiet is the first completed long-term randomised controlled trial focusing on prodromal Alzheimer's disease defined according to the International Working Group (IWG-1) criteria. Benefit was observed in relevant secondary cognitive-functional and brain atrophy outcome measures, but not in the primary neuropsychological test battery and other secondary measures including dementia diagnosis. Our findings support the hypothesis that intervening early in the disease continuum might achieve benefits more readily than late intervention.

**Implications of all the available evidence**

Our results emphasise the difficulty in finding adequately sensitive outcome measures for trials in prodromal Alzheimer's disease. The potential for impact on disease progression, combined with the feasibility aspects including the observed high long-term compliance, moderate costs of the intervention, the potentially relative ease of implementation in clinical practise, as well as the clear need for treatment, warrant further research on multinutrient intervention in early Alzheimer's disease.

## Methods

### Study design and participants

The LipiDiDiet study was a 24-month randomised, controlled, double-blind, parallel-group, multicentre trial done in 11 study sites in Finland, Germany, the Netherlands, and Sweden (appendix) with one to four optional, 12-month, double-blind extension periods. Participants were primarily recruited from memory clinics and had routine assessments in the year before screening. The study was completed as planned. Here we report 24-month findings; extension studies are currently ongoing and will be reported later. We enrolled participants aged 55–85 years with a Mini-Mental State Examination (MMSE) score of 24 points or higher (≥20 if education level ≤6 years) who fulfilled criteria for prodromal Alzheimer's disease^3^ as defined by episodic memory disorder (performance below one standard deviation on two of eight cognitive tests [at least one on memory]) and evidence for underlying Alzheimer's disease pathology based on positive findings from at least one of the following diagnostic tests: CSF, MRI, and ^18^F fluorodeoxyglucose (^18^F-FDG) PET analysis (full list of inclusion criteria is in the appendix). We excluded participants with dementia according to Diagnostic and Statistical Manual of Mental Disorders, 4th Edition (DSM-IV); historical use of donepezil, rivastigmine, galantamine, or memantine, use of omega-3 preparations, alcohol or drug abuse, major depressive disorders (DSM-IV) or other concomitant serious conditions, intake of vitamins B6, B12, folic acid, vitamin C, or vitamin E of more than 200% of the recommended daily intake, those who participated in any other clinical trial in the last 30 days, and with an MRI or CT scan consistent with a diagnosis of stroke, intracranial bleeding, mass lesion, or normal pressure hydrocephalus (minimal white matter changes and up to two lacunar infarcts judged to be clinically insignificant were allowed). Participants who progressed to dementia during the trial could remain in the trial and start approved Alzheimer's disease medication, according to the clinician's judgment. The protocol was amended to allow participants who progressed to dementia to switch to the active product after it became generally available (appendix). The study protocol and consent forms were approved by the local ethical committees of all participating sites, and all participants provided written informed consent before study participation. The study was done in accordance with the Declaration of Helsinki and International Conference on Harmonization Good Clinical Practice guidelines.

### Randomisation and masking

Eligible participants were randomly assigned (1:1) to receive either the active or control product once daily according to a randomisation list, which was computer generated by Nutricia Research, stratified by site, and in block sizes of four. Sealed opaque envelopes were available for each participant. After acceptance of a participant to the trial, the envelope with the lowest unused number was opened at the site, containing the code for that participant. The active and control products were isocaloric and similar in appearance and flavours (vanilla and strawberry). All study personnel and participants, including the investigators and study-site staff, were masked to treatment assignment. Only the trial-independent statistician and the independent data monitoring committee, who reviewed interim data for safety and efficacy purposes, were partially unmasked.

### Procedures

We enrolled eligible participants at a combined screening and baseline visit or during a separate baseline visit. Efficacy evaluations were done at baseline, 6, 12, and 24 months (appendix). Study sites received training on outcome assessments. Visits to the study nurse or physician were scheduled every 3 months during the first year and every 6 months thereafter. To maintain motivation, check compliance, and monitor safety, participants were contacted by phone throughout the trial (once per month during the first 6 months and every 2 months thereafter). Study products were dispensed to the participants every 3 months. Participants in the active group were given the medical food Souvenaid, a 125 mL once-a-day drink containing the specific nutrient combination Fortasyn Connect (appendix). Participants in the control group were given a 125 mL once-a-day control drink. The study product was produced by Nutricia (Zoetermeer, the Netherlands).

### Outcomes

The primary efficacy endpoint was the change over 24 months in a composite score of cognitive performance based on a neuropsychological test battery (NTB; appendix),^29^ assessed by study neuropsychologists at each site at baseline and months 6, 12, and 24. Based on advances in Alzheimer's disease research and results from a clinical trial with the active product,^24^ protocol amendments were made after the study started and before database lock to specify the composite scores of the NTB and to limit the number of secondary endpoints (appendix). The NTB primary endpoint was a composite *Z* score based on Consortium to Establish a Registry for Alzheimer's disease (CERAD) 10-word list learning immediate recall, CERAD 10-word delayed recall, CERAD 10-word recognition, category fluency, and letter digit substitution test (LDST). Secondary endpoints were NTB memory domain (composite *Z* score based on CERAD 10-word list learning immediate recall, delayed recall, and recognition), NTB executive function domain (composite *Z* score based on category fluency, Wechsler Memory Scale revised digit span total score, concept shifting test condition C [corrected for the zero trials], and LDST), and NTB total (composite *Z* score based on all 16 items of the NTB). Composite scores were calculated as *Z* scores standardised to the baseline mean and SD, with higher scores suggesting better performance. Other secondary endpoints, assessed at baseline, month 12, and month 24, unless stated otherwise, were change from baseline over 24 months in clinical dementia rating-sum of boxes (CDR-SB), brain volumes based on MRI (three-dimensional T1-weighted anatomical scans of total hippocampal, whole-brain, and ventricular volumes; details of MRI acquisition and central analysis are in the appendix), progression to dementia (according to criteria defined by DSM-IV, the National Institute of Neurological and Communicative Disorders and Stroke, and the Alzheimer's Disease and Related Disorders Association criteria for Alzheimer's disease), serum concentrations of HDL cholesterol and LDL cholesterol, plasma fatty acids (DHA and EPA, assessed at baseline and months 3, 6, 12, and 24; for laboratory analysis, see appendix), and DHA in CSF (CSF analysis not yet finalised). Safety assessments included adverse events, use of concomitant medications, consumption of nutritional supplements, study product compliance, vital signs (heart rate, systolic blood pressure, and diastolic blood pressure), and clinical safety laboratory tests. To monitor product compliance, we asked participants to record the amount of study product taken in a daily diary, which was collected at each visit. Study product compliance was defined as the percentage of study product used throughout the study period compared with the prescribed dosage. Compliance was calculated only for participants who completed the study product diary for at least 75% of their actual study time. An additional sensitivity calculation was done to include all available data up to the start of rescue medication (defined as use of active product or approved Alzheimer's disease medication after progression to dementia). In both calculations, missing diary intake entries were assumed to be 0. We coded adverse events with the Medical Dictionary for Regulatory Activities (version 18.0).

### Statistical analyses

Based on a *t* test and 5% significance level, we calculated that a sample size of 300 randomly assigned participants would be sufficient to provide 90% power to detect a 40% difference in NTB score change between groups at the end of the study. Based on results from a study in patients with mild Alzheimer's disease dementia,^29^ we expected the NTB *Z* score in the control group to decrease by −0·4 (SD 0·4) during 24 months. The sample size allowed for 20% dropout. We did a prespecified, blinded re-estimation of the SD to assess the adequacy of the calculated sample size. Representatives of the LipiDiDiet Consortium reviewed the SDs calculated from the interim dataset and concluded that they matched the estimated SDs in the protocol. Additionally, we amended the protocol to do an interim analysis for safety (occurrence of adverse events) and efficacy after approximately a third of participants completed the study. Between-group analyses on partially unmasked data were done by the trial-independent statistician and results were reviewed by the independent data monitoring committee, which recommended continuation of the study without modification.

We obtained NTB composite *Z* scores by averaging the individual NTB items' *Z* scores and weighting according to the number of NTB items available. The minimum number of NTB items required was set to four of five for NTB primary endpoint, three of three for NTB memory domain, three of four for NTB executive function domain, and 12 of 16 for NTB total. Analyses were done on the modified intention-to-treat (mITT) population of all participants randomly assigned, excluding data after the start of rescue medication. We did per-protocol analyses on all participants from the mITT population, excluding the respective visits of participants with major protocol deviations defined during a data review of masked data. The most common reason for exclusion from the per-protocol analysis was substantial irregular study product intake (appendix). All randomised participants who consumed at least one dose of study product were included in safety analyses. To allow for separate evaluation of safety data collected before and after a switch to active study product after progression to dementia, analyses were done in two safety phases: the double-blind treatment phase and active treatment phase.

We analysed the primary endpoint and all secondary endpoints of a continuous type as prespecified in the statistical analysis plan, using a linear mixed model for longitudinal data with change from baseline as the response variable and linear time (days since baseline), baseline score, randomised treatment, and time × treatment as fixed effects. This is a multi-level model with three levels: measurements, participants, and sites. We used a random intercept with a variance components covariance structure within sites (small sites were pooled within country) and a random intercept and slope for time with an unstructured covariance structure within participants. Other covariance structures could be applied in case of converging issues. This model's estimated difference between the active and control groups in terms of mean change from baseline at month 24 was used as the primary indication of treatment effect during the 24-month intervention period. A baseline measurement or characteristic that showed an imbalance between the groups and was found to be a significant predictor for outcome parameters was considered a prognostic factor and included in the statistical models. We did a planned sensitivity analysis using a mixed model for repeated measures with change from baseline as the response variable and time as categorical (planned visit), baseline score, randomised treatment, and time × treatment as fixed effects. In this analysis, treatment effects were evaluated for each timepoint separately without assuming a treatment effect that increases linearly over time. We did an additional sensitivity analysis on participants who completed the 24-month study by using an analysis of covariance completer analysis with change from baseline as outcome, treatment as fixed factor, and baseline score as a covariate. We did an additional sensitivity analysis taking into account missing data due to dropout using a joint model. The joint model combined a mixed model comparable to our original model (mixed model) with a Cox proportional hazards model for time to dropout (appendix). We did a predefined subgroup analysis in participants with MMSE score 26 or higher at baseline using the same statistical models as for the primary and secondary endpoints. We aligned rules for visit windows between the statistical models to allow a visit window of 3 months (before and after scheduled visit date) for all visits.

p values of less than 0·05 were deemed statistically significant in comparisons of efficacy and safety data. Statistical analyses were done with SAS software (version 9.4). The study is registered with the Dutch Trial Register, number NTR1705.

### Role of the funding source

The funder of the study had no role in study design, data collection, data analysis, data interpretation, or writing of the report. HS, AS, PJV, SBH, KB, MK, and TH had full access to all the data in the study. The corresponding author had final responsibility for the decision to submit for publication.

## Results

**Figure 1:**
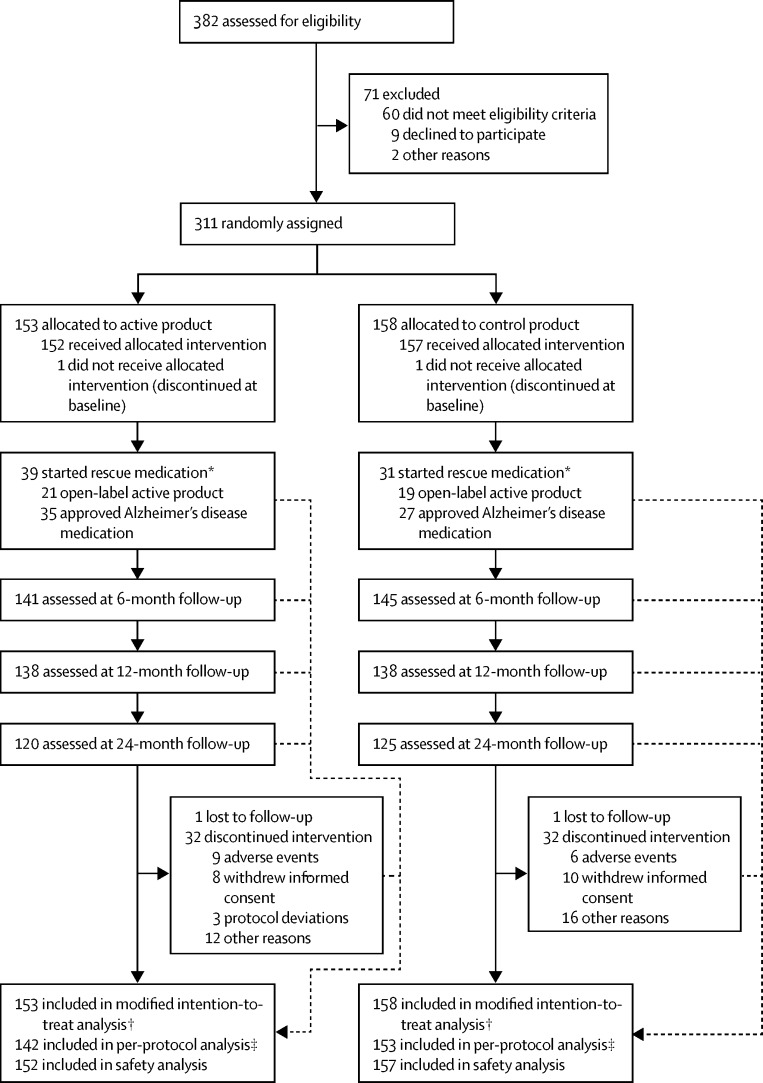
Trial profile *Rescue medication was defined as the use of active product or approved Alzheimer's disease medication after progression to dementia. †All randomly assigned participants, excluding visit data after the start of rescue medication. ‡Respective visits of participants were additionally excluded in cases of major protocol deviations; number based on participants with at least one follow-up visit in the per-protocol dataset.

Between April 20, 2009, and July 3, 2013, 311 participants of 382 screened were randomly assigned to either the active group (n=153) or the control group (n=158; figure 1). Dropout was 22% in the active group and 21% in the control group (p=0·891, Fisher's exact test), with no significant difference in time to dropout between groups. The main reasons for dropout were adverse events (n=15; appendix), withdrawal of informed consent (n=18), or other reasons (n=28). Five participants died during the study: four in the active group due to respiratory failure (n=2), bronchial carcinoma (n=1), and infection (n=1); and one in the control group due to sudden death with no apparent cause. All deaths were assessed as not related to the study product. Mean age was 71·0 years, and 154 (50%) of 311 participants were men. Further baseline characteristics of study participants are shown in table 1. Results and parameters used in assessment of eligibility for prodromal Alzheimer's disease at screening are summarised in the appendix, including classification according to the International Working Group (IWG)-1, IWG-2, and National Institute of Aging-Alzheimer Association (NIA-AA) criteria.^3^, ^4^, ^5^ The active and control groups were similar at baseline (table 1), except for MMSE score. Baseline MMSE was also found to be a significant predictor of outcome parameters, which made it a potential prognostic factor; therefore, it was included as a covariate in all statistical models except MMSE subgroup analyses.

**Table 1:** Baseline characteristics

		**Control (n=158)**	**Active (n=153)**
Age (years)			
	Mean (SD)	70·7 (6·2)	71·3 (7·0)
	Median (range)	71 (52–84)	72 (50–86)
Sex			
	Men	73 (46%)	81 (53%)
	Women	85 (54%)	72 (47%)
Ethnic origin			
	White	157 (99%)	152 (99%)
	Black	0 (0)	1 (1%)
	Other	1 (1%)	0 (0)
Education (years)		10·7 (3·6)	10·6 (3·9)
Mini-Mental State Examination		26·9 (1·9)	26·4 (2·1)
*APOE* ɛ4 genotype*			
	Carrier	90/143 (63%)	83/138 (60%)
	Non-carrier	53/143 (37%)	55/138 (40%)
Cognitive measures (composite *Z* score)			
	NTB primary endpoint	0·00 (0·68)	−0·00 (0·70)
	NTB memory domain	0·03 (0·82)	−0·02 (0·87)
	NTB executive function domain	−0·01 (0·71)	0·01 (0·71)
	NTB total	−0·02 (0·56)	0·02 (0·57)
CDR-SB		1·75 (1·14)	1·87 (1·17)
MRI brain volumes (cm^3^)†			
	Total hippocampal volume	5·70 (1·25)	5·62 (1·10)
	Whole brain volume	1377·30 (84·08)	1370·56 (81·64)
	Ventricular volume	53·95 (25·31)	58·35 (26·66)
CSF†			
	Aβ_42_ (pg/mL)	401·1 (196·1)	426·9 (292·7)
	(Aβ_42_/Aβ_40_) × 10	0·62 (0·25)	0·65 (0·29)
	Total tau (pg/mL)	634·8 (287·7)	591·9 (260·9)
	Phosphorylated tau (pg/mL)	80·3 (30·6)	74·2 (25·8)

Data are mean (SD), n (%), n/N (%), or median (range). NTB=neuropsychological test battery. CDR-SB=clinical dementia rating sum of boxes. Aβ=amyloid β.

* Data not available for all randomised participants. Percentages are calculated based on number of participants with available data.

† Central analysis CSF data available for n=107 and MRI data for n=279 (appendix).

Primary and main secondary endpoints are reported in table 2, in which higher scores indicate better performance for all endpoints except for CDR-SB and ventricular volume. Mean change from baseline to month 24 in the NTB primary endpoint was −0·108 (SD 0·528) in the control group, and −0·028 (SD 0·453) in the active group. The decline in the control group was lower than the prestudy estimate of −0·4 during 24 months. There was no statistically significant difference between groups for the primary endpoint (estimated mean treatment difference of 0·098, 95% CI −0·041 to 0·237; p=0·166). Similarly, there were no statistically significant differences when the analyses were done without adjustment for baseline MMSE (appendix) and in the sensitivity analysis (table 2).

**Table 2:** Primary endpoint and main secondary endpoints

		**Control (n=158)**		**Active (n=153)**		**Difference**	Mixed model*, **p value**	**Sensitivity analysis**†**p value**	**Effect size**‡**Cohen's *d***
		Mean (SD)§	n	Mean (SD)§	n	Estimate (95% CI)¶			
**Primary endpoint**									
NTB primary endpoint (*Z* score)									
	Modified intention-to-treat	−0·108 (0·528)	141	−0·028 (0·453)	134	0·098 (−0·041 to 0·237)	0·166	0·214	0·17
	Per-protocol	−0·122 (0·570)	123	0·045 (0·414)	116	0·140 (−0·017 to 0·296)	0·080	0·043	0·24
**Secondary endpoints**									
NTB memory domain (*Z* score)									
	Modified intention-to-treat	−0·130 (0·619)	140	0·003 (0·569)	134	0·138 (−0·027 to 0·303)	0·101	0·112	0·17
	Per-protocol	−0·151 (0·663)	122	0·083 (0·532)	116	0·181 (−0·005 to 0·367)	0·057	0·026	0·25
NTB executive function domain (*Z* score)									
	Modified intention-to-treat	−0·039 (0·506)	141	−0·145 (0·445)	133	−0·043 (−0·180 to 0·095)	0·541	0·281	−0·08
	Per-protocol	−0·045 (0·546)	123	−0·090 (0·381)	115	0·009 (−0·137 to 0·155)	0·906	0·854	0·01
NTB total (Z score)									
	Modified intention-to-treat	−0·059 (0·400)	140	−0·047 (0·347)	134	0·027 (−0·078 to 0·132)	0·612	0·729	0·07
	Per-protocol	−0·061 (0·419)	122	−0·006 (0·317)	116	0·058 (−0·056 to 0·172)	0·316	0·352	0·15
CDR-SB||									
	Modified intention-to-treat	1·12 (1·72)	119	0·56 (1·32)	111	−0·60 (−1·01 to −0·19)	0·005	0·004	0·33
	Per-protocol	1·07 (1·82)	98	0·40 (1·13)	94	−0·72 (−1·16 to −0·28)	0·002	0·002	0·43
MRI total hippocampal volume (cm^3^)									
	Modified intention-to-treat	−0·43 (0·33)	104	−0·30 (0·27)	96	0·12 (0·04 to 0·21)	0·005	0·005	0·22
	Per-protocol	−0·42 (0·32)	90	−0·28 (0·28)	86	0·12 (0·03 to 0·21)	0·010	0·008	0·20
MRI whole brain volume (cm^3^)									
	Modified intention-to-treat	−24·24 (20·93)	90	−20·27 (17·79)	83	3·66 (−2·81 to 10·14)	0·265	0·284	0·21
	Per-protocol	−23·88 (19·90)	77	−17·89 (16·88)	73	5·04 (−2·02 to 12·10)	0·160	0·137	0·29
MRI ventricular volume (cm^3^)||									
	Modified intention-to-treat	7·80 (5·53)	106	5·96 (4·66)	94	−1·36 (−2·70 to −0·03)	0·046	0·042	0·22
	Per-protocol	7·40 (4·79)	92	5·39 (4·50)	83	−1·40 (−2·79 to −0·02)	0·046	0·042	0·20

n=number of participants with at least one post-baseline value in the mixed model. p values are for effect of intervention over 24 months. NTB=neuropsychological test battery. CDR-SB=clinical dementia rating sum of boxes. MMSE=Mini-Mental State Examination.

* Mixed model: linear mixed model for longitudinal data with change from baseline as outcome, baseline score, and baseline MMSE as covariates, and real measurement time as a continuous variable.

† Sensitivity analysis: mixed model for repeated measures with change from baseline as outcome, baseline score, and baseline MMSE as covariates, and planned visit time as a categorical variable.

‡ Cohen's *d* standardised effect size calculated based on the mean treatment difference over 24 months as estimated in the mixed model and the pooled SD; results are presented so that a positive effect size indicates improved performance in the active versus control group and vice versa.

§ Data for active and control groups are presented as observed mean change from baseline at month 24 (SD).

¶ Difference (active minus control) is calculated as based on least squares means for change from baseline over 24 months as estimated in the mixed model.

|| Higher scores indicate worse performance; for all other endpoints, higher scores indicate better performance.

**Figure 2:**
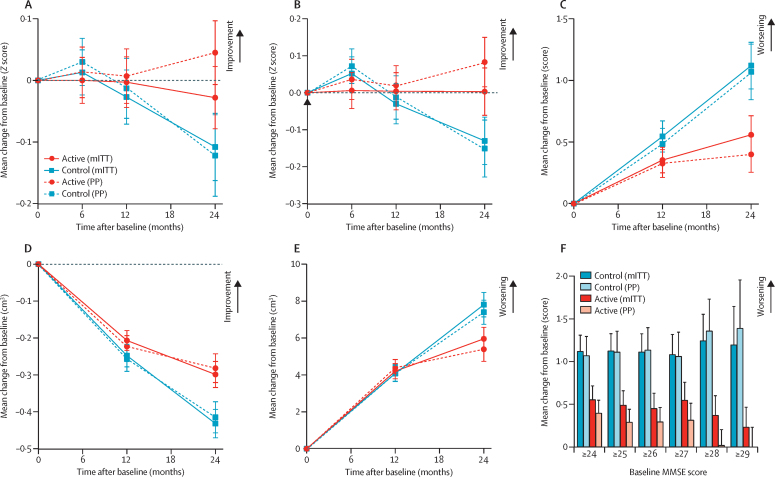
Changes in main endpoints during the 24-month intervention (A) NTB primary endpoint. (B) NTB memory domain. (C) CDR-SB. (D) MRI total hippocampal volume. (E) MRI ventricular volume. (F) CDR-SB in subgroups defined by baseline MMSE. Data are observed mean change from baseline; error bars are SE. Sample size by baseline MMSE subgroup (control/active): ≥24: mITT 117/106 (PP 96/89), ≥25: 104/91 (86/75), ≥26: 95/79 (78/66), ≥27: 77/63 (66/53), ≥28: 55/43 (48/37), ≥29 29/21 (24/19). CDR-SB=clinical dementia rating-sum of boxes. mITT=modified intention-to-treat analysis. MMSE=Mini-Mental State Examination. NTB=neuropsychological test battery. PP=per-protocol analysis.

No statistically significant differences were observed for the secondary NTB composite scores. For CDR-SB, there was significantly less worsening in the active group than in the control group during 24 months (p=0·005; table 2, figure 2C). Similar results were obtained without adjustment for baseline MMSE (appendix) and in the sensitivity analysis (table 2). The worsening in CDR-SB was 45% less in the active group than in the control group, based on the estimated change from baseline over 24 months.

We observed significantly less reduction in hippocampal volume (p=0·005) and less increase in ventricular volume (p=0·046) during 24 months in the active group than in the control group (table 2, figure 2D, 2E). The rates of deterioration were lower in the active group than in the control group, both for hippocampal (26%) and ventricular volumes (16%). Sensitivity analyses confirmed these observations for hippocampal volume and ventricular volume. No statistically significant differences between groups were found for changes in whole brain volume (table 2).

During the 24-month trial period, 59 (37%) participants in the control group and 62 (41%) in the active group were diagnosed with dementia (p=0·642, Fisher's exact test; appendix). HDL cholesterol concentration significantly increased in the active group compared with the control group, but absolute changes were very small (<5%), and no differences between groups were found for changes in LDL cholesterol over 24 months (appendix).

Differences in cognition-related scores between groups were more pronounced in per-protocol analyses than in the mITT analyses, particularly for the NTB primary endpoint and the NTB memory domain (table 2; appendix). There were no differences in baseline characteristics between active and control groups among participants included in the per-protocol analyses (appendix). Forest plots showing an overview of the mITT and per-protocol results from the different statistical models are provided in the appendix.

Predefined subgroup analyses (MMSE score ≥26) in mITT and per-protocol populations are shown in the appendix. We observed statistically significant differences between groups for CDR-SB and hippocampal volume in the mITT population, and for the NTB primary endpoint (mixed model p=0·131, sensitivity analysis p=0·044) and NTB memory domain (mixed model p=0·073, sensitivity analysis p=0·017) in the per-protocol population. Baseline MMSE was an effect modifier for CDR-SB in the per-protocol population (mixed model p=0·053 for interaction term treatment effect × baseline MMSE). Therefore, we did an exploratory analysis of CDR-SB performance across the spectrum of baseline MMSE (≥24 to ≥29), which suggested that the treatment effect on CDR-SB increased with higher baseline MMSE scores (figure 2F; appendix).

Self-reported adherence to the intervention was high, both when calculated in all participants (mean 93·4% [SD 8·8] in both groups) and when calculated using all available data in the mITT (excluding data collected after starting rescue medication: mean 87·3% [SD 22·9] in active and 86·8% [23·4] in control). This adherence was confirmed by significant biochemical changes in plasma DHA and EPA during 24 months in the active group compared with no changes in the control group (p<0·0001; appendix). The incidences of adverse events and serious adverse events were similar between groups (p=0·864 and p=0·487; table 3), and among the 66 participants who dropped out (active *vs* control: 24 [73%] *vs* 22 [67%], p=0·789 and 8 [24%] *vs* three [9%], p=0·185). None of the serious adverse events were regarded as related to the study product and dropout due to adverse events was not significantly different between groups (nine [6%] in the active group *vs* six [4%] in the control group, p=0·437).

**Table 3:** Summary of adverse events in all participants who were randomly assigned and on double-blind treatment

	**Control (n=157)**	**Active (n=152)**
**All events**		
At least one adverse event	138 (88%)	132 (87%)
At least one serious adverse event	30 (19%)	34 (22%)
Most common serious adverse events*		
Myocardial infarction	2 (1%)	0 (0)
Fall	1 (1%)	2 (1%)
Intervertebral disc protrusion	2 (1%)	0 (0)
Osteoarthritis	3 (2%)	0 (0)
Syncope	0 (0)	3 (2%)
(Major) depression	3 (2%)	1 (1%)
Cardiac operation	2 (1%)	0 (0)
Hospitalisation	0 (0)	2 (1%)
Circulatory collapse	0 (0)	2 (1%)
**Most common adverse events**†		
Vertigo	12 (8%)	6 (4%)
Diarrhoea	14 (9%)	7 (5%)
Cystitis	9 (6%)	4 (3%)
Nasopharyngitis	16 (10%)	7 (5%)
Respiratory tract infection	9 (6%)	7 (5%)
Urinary tract infection	9 (6%)	7 (5%)
Fall	8 (5%)	11 (7%)
Arthralgia	9 (6%)	4 (3%)
Back pain	5 (3%)	10 (7%)
Headache	12 (8%)	9 (6%)
Cough	10 (6%)	2 (1%)

Data are n (%). Adverse events are presented by Medical Dictionary for Regulatory Activities preferred term.

* Only those reported by at least two participants in either group are shown.

† Only those reported by at least 5% of participants in either group are shown.

## Discussion

Prodromal Alzheimer's disease is a new area of Alzheimer's disease research, with clinical research practices still under development. LipiDiDiet is the first randomised, controlled, double-blind, multicentre, international trial of a non-pharmacological intervention in prodromal Alzheimer's disease. No significant difference was found between groups for the NTB primary endpoint in the mITT analysis or on conversion to dementia. However, there was some evidence of a beneficial effect of the multinutrient intervention at the cognitive-functional level (detected by CDR-SB) and ameliorated structural brain changes (hippocampal and ventricular volume) shown on MRI scans.

The LipiDiDiet study was initiated shortly after the first criteria for prodromal Alzheimer's disease were published.^3^ It has since become clear that changes in cognitive performance with currently used tests are not very pronounced in early Alzheimer's disease during time intervals close to 2 years.^30^, ^31^ The study design was based on a previous 12-month trial in Alzheimer's disease dementia;^29^ however, in our study, the control group had only a quarter of the projected 24-month decline on the NTB primary endpoint, possibly because of the earlier disease stage of the pre-dementia participants than those with Alzheimer's disease dementia. The lower than expected decline is in agreement with the previous observations of limited cognitive changes over 2 years. The small cognitive decline on the NTB primary endpoint in the control group was mainly due to stable performance during the first year of intervention, followed by a steeper decline in the second year. Conversely, CDR-SB scores had already significantly declined at month 12 in the control group. Therefore, in mITT analyses, the significant benefit on CDR-SB was combined with an absence of clear effects on NTB cognition endpoints, although benefits on the NTB primary endpoint and the NTB memory domain were suggested in the per-protocol analyses. Notably, the main reason for exclusion from the per-protocol analysis was no or irregular intake of study product, which emphasises the importance of sustained intake, as observed previously.^27^, ^32^

The effect on CDR-SB in the LipiDiDiet trial differs from previous mild Alzheimer's disease dementia trials by adding a benefit at the cognitive-functional level.^23^, ^24^ The longer treatment duration and intervention at an earlier disease stage than in the previous dementia trials might be important reasons for this observation. The apparently more pronounced stabilisation of CDR-SB scores with increasing baseline MMSE observed in the active group indicates that early rather than late treatment within the prodromal stage might support better outcome with this cognitive-functional measure, in line with previous results from Fortasyn Connect trials,^23^, ^24^, ^26^ which showed that earlier intervention might increase the benefit. Within the disease continuum, early intervention might also be an important contributory factor to the similarity in progression to dementia in both treatment groups, because participants at baseline were only 2 years or less from advancing to dementia and therefore treatment efficacy might not have been sufficient to translate into less conversion to dementia. Moreover, dementia diagnosis was clustered at major study visits. Dementia diagnosis is a dichotomisation of the decline continuum, whereas CDR-SB is a sensitive measure of decline across a continuous scale. Therefore, CDR-SB might better reflect disease progression along our entire prodromal population.

No cognitive test is generally accepted as the gold standard for trials in prodromal Alzheimer's disease, although research has highlighted the potential usefulness of composite measures.^33^ Use of compound cognitive test batteries such as NTB combining performance on different validated tests have been suggested to help detection of more subtle changes that occur in pre-dementia disease stages.^34^ In the meantime, preliminary guidelines from regulatory agencies emphasise the importance of establishing the clinical value of treatment and suggest using a combined cognitive-functional measure such as CDR-SB, which showed reliability and validity in prodromal Alzheimer's disease and mild cognitive impairment due to Alzheimer's disease, and proposed CDR-SB as a single primary endpoint for efficacy.^35^, ^36^ However, this information was not available at the time of our trial design.

Although it is generally difficult to translate performance on cognitive tests into clinical benefits, the CDR-SB is built on real-life items such as handling household emergencies, handling financial transactions, and forgetting a major event, which facilitates assessment of clinical benefit. For early Alzheimer's disease, a reduction by 0·5 or 1·0 in CDR-SB was proposed to capture both efficacy and clinical relevance.^1^ The current emphasis on using more sensitive cognitive or functional measures in ongoing trials in prodromal Alzheimer's disease is a major shift from the previous focus on progression to dementia used in unsuccessful trials in mild cognitive impairment.^33^

In addition to the CDR-SB cognition-function benefit, we observed benefits on progression of structural changes in the brain. The hippocampus is affected early in Alzheimer's disease, and the rate of hippocampal atrophy over time is considered a reliable measure of Alzheimer's disease progression.^4^ We noted 26% less reduction in hippocampal volume in the active group compared with the control group and the active group also had 16% less increase in ventricular volume, suggesting an interaction of the treatment with the disease process. Interaction could be hypothesised based on animal and mild cognitive impairment studies that showed effects on Alzheimer's disease-related brain pathologies.^9^, ^10^

Prodromal Alzheimer's disease was defined according to the IWG-1 criteria.^3^ Currently, three sets of research criteria are available for Alzheimer's disease diagnosis in people with mild cognitive impairment: IWG-1,^3^ IWG-2,^4^ and NIA-AA^5^ criteria. Comparative analysis showed that all three predict cognitive decline with reasonable accuracy.^37^ Baseline characteristics in our study were as expected for a prodromal Alzheimer's disease population, including the CSF biomarker profile; percentage of *APOE* ε4 carriers; and IWG-1, IWG-2, and NIA-AA criteria (appendix). The main differences between the IWG-1 and IWG-2 criteria are the definition of in-vivo evidence of Alzheimer's disease pathology (medial temporal lobe atrophy on MRI is included in IWG-1, but not in IWG-2) and the clinical Alzheimer's disease phenotypes (IWG-1 focuses on a typical amnestic phenotype, whereas IWG-2 also includes atypical, non-amnestic phenotypes). Thus, we cannot make inferences on intervention effects in prodromal Alzheimer's disease with atypical, non-amnestic phenotypes. This study indicates that populations within prodromal Alzheimer's disease exist who might benefit differently from early intervention. Baseline MMSE scores and decline in cognitive function contributed to different levels of benefit; further currently unknown factors might contribute as well. Identification of those individuals could aid in the ongoing process of refining prodromal Alzheimer's disease definition and prodromal Alzheimer's disease clinical trial design.

As expected from previous trials,^23^, ^24^, ^26^ study product compliance was high, and adverse events and serious adverse events were consistent with the studied population and the known safety profile of the active product.^23^, ^24^, ^26^ The proportion of participants with at least one serious adverse event (34 [22%] in the active group and 30 [19%] in the control group) and percentage of dropouts due to adverse events (nine [6%] in the active group and six [4%] in the control group) in our study are in the same range as those reported by Coric and colleagues^38^ for the control group (31 [23.7%] patients with at least one serious adverse event and 13 [9.9%] dropouts due to adverse events).

Our study has some limitations. First, cognitive decline in this prodromal Alzheimer's disease population was much lower than expected, rendering the primary endpoint inadequately powered. Therefore, future trials aiming to implement this NTB endpoint might benefit from larger sample sizes and a longer duration of intervention than used in our study, or a cognitive composite designed for this pre-dementia population. Second, our 24-month trial was not designed with progression to dementia as a primary focus, thereby limiting the ability to draw conclusions on this outcome. Additionally, use of MRI as an alternative to CSF or PET amyloid assessments might have resulted in a somewhat more heterogeneous group of participants, because medial temporal atrophy on MRI can be both amyloid-related and non-amyloid-related.^4^ Finally, we included a demographically restricted population, largely comprising white participants from central European countries and Scandinavia. Participants completing the 24-month intervention were eligible to continue in the double-blind extension studies, which will provide additional data on long-term efficacy.

In conclusion, the multinutrient intervention had no significant effect on the NTB primary endpoint over 2 years in prodromal Alzheimer's disease, although potential benefits were seen on the cognitive-functional measure CDR-SB and brain atrophy measures. Further investigation of multinutrient approaches in early Alzheimer's disease stages is warranted.
