# Supplementary material for: 24-month intervention with a specific multinutrient in people with prodromal Alzheimer's disease (LipiDiDiet): a randomised, double-blind, controlled trial
Source: Lancet Neurol. 2017 Dec;16(12):965–75. doi: 10.1016/S1474-4422(17)30332-0 (PMC5697936; doi:10.1016/S1474-4422(17)30332-0)
Supplement: Supplementary appendix [file mmc1.pdf]

THE LANCET
Neurology

Supplementary appendix

This appendix formed part of the original submission and has been peer reviewed. We post it as supplied by the authors.

Supplement to: Soininen H, Solomon A, Visser PJ, et al, on behalf of the LipiDiDiet clinical study group. 24-month intervention with a specific multinutrient in people with prodromal Alzheimer's disease (LipiDiDiet): a randomised, double-blind, controlled trial. *Lancet Neurol* 2017; published online Oct 30. http://dx.doi.org/10.1016/S1474-4422(17)30332-0.

Supplementary appendix

Supplement to: Soininen H, et al. 24-month intervention with a specific multinutrient in patients with prodromal Alzheimer's disease (LipiDiDiet): a randomised, double-blind, controlled trial

Table of contents

LipiDiDiet clinical study group 2
- Collaborators 2
- LipiDiDiet Trial Steering Committee 2

Additional methodological details 3
- Study protocol 3
- Eligibility criteria 3
- Neuropsychological test battery (NTB) 3
- MRI acquisition and analyses 4
- Biochemical analyses 5
- Storage conditions of study product 5
- Relevant protocol amendments: changes to methods and trial outcomes after start of the trial 5
- Definition of the per-protocol population 6
- Joint model analyses and results 6

References 7

Additional tables and figures 8
- Supplementary table 1: Nutritional composition of Fortasyn Connect, the specific nutrient combination in the medical food Souvenaid 8
- Supplementary table 2: Parameters used to assess eligibility for prodromal Alzheimer's disease at screening . 9
- Supplementary table 3: Classification according to the IWG-1, IWG-2, and NIA-AA criteria based on centrally analysed CSF and MRI data 10
- Supplementary table 4: Distribution of baseline MMSE by treatment group (ITT) 11
- Supplementary table 5: Primary endpoint and main secondary endpoints, unadjusted for baseline MMSE 12
- Supplementary table 6: Predefined subgroup analysis for participants with baseline MMSE ≥26 13
- Supplementary table 7: Other secondary endpoints 14
- Supplementary table 8: Progression to dementia (ITT) 14
- Supplementary table 9: Baseline characteristics of the per-protocol population 15
- Supplementary table 10: CDR-SB in subgroups defined by baseline MMSE 16
- Supplementary table 11: Observed means (SD) by visit for primary endpoint and main secondary endpoints (mITT) 17
- Supplementary table 12: Listing of adverse events contributing to study discontinuation 18
- Supplementary figure 1: Forest plots 19

LIPIDIDIET CLINICAL STUDY GROUP

Collaborators

Finland

- University of Eastern Finland, Kuopio (Hilkka Soininen*, Ilona Hallikainen, Merja Hallikainen, Seppo Helisalmi, Tarja Lappalainen, Yawu Liu, Teemu Paajanen)

Sweden

- Karolinska University Hospital, Stockholm (Miia Kivipelto*, Alina Solomon, Lars-Olof Wahlund, Yvonne Freund-Levi, Niels Andreasen, Göran Hagman, Stina Lindblom)
- University of Gothenburg, Gothenburg (Kaj Blennow)

Germany

- Saarland University, Homburg (Tobias Hartmann, Klaus Fassbender*, Matthias Riemenschneider*, Marcus OW Grimm, Aline Klees-Rollmann, Maxine Luley, Epameinondas Lyros, Robert Schomburg, Jennifer Kennel, Daniela Ramelli)
- Central Institute of Mental Health, Mannheim (Lutz Frölich*, Lucrezia Hausner)
- University Hospital of Tübingen, Tübingen (Christoph Laske*, Thomas Leyhe*, Christian Mychajliw, Niklas Koehler)
- University Hospital Regensburg, Regensburg (Stephan Schiekofer*, Hans Klünemann*)
- Heidelberg University Hospital, Heidelberg (Johannes Schröder*)
- Institute for Clin. Chem. and Clin. Pharm., Universitätsklinikum Bonn, Bonn (Dieter Lütjohann)

The Netherlands

- VU University Medical Center, Amsterdam (Pieter Jelle Visser*, Philip Scheltens, Ineke van Rossum, Nienke Scheltens, Daniela Bertens, Mara ten Kate, Frederik Barkhof)
- Zuwe Hofpoort Hospital, Woerden (Johanna ML Henselmans*)
- St. Elisabeth Hospital, Tilburg (Gerwin Roks*)
- Nutricia Research, Nutricia Advanced Medical Nutrition, Utrecht (Anneke MJ van Hees)

USA

- Pentara Corporation, Salt Lake City, UT, USA (Suzanne B Hendrix, Noel Ellison)

*Principal investigator.

LipiDiDiet Trial Steering Committee

- Tobias Hartmann (LipiDiDiet project coordinator), Saarland University, Homburg, Germany
- Hilkka Soininen (clinical work package leader / coordinating investigator), University of Eastern Finland, Kuopio, Finland
- Miia Kivipelto, Karolinska University Hospital, Stockholm, Sweden
- Alina Solomon, Karolinska University Hospital, Stockholm, Sweden
- Pieter Jelle Visser, VU University Medical Center, Amsterdam, the Netherlands

ADDITIONAL METHODOLOGICAL DETAILS

Study protocol

The study protocol is available online: http://lipididiet.eu/fileadmin/lipididiet/publications/LipiDiDietStudyProtocol.pdf.

Eligibility criteria

Inclusion criteria

- Prodromal Alzheimer's disease (AD) as defined by A) episodic memory disorder and B) evidence for underlying AD pathology.
  A) Episodic memory disorder is defined as -1 standard deviation on 2 out of 8 tests, at least 1 memory:
  Memory
    - Free and cued selective reminding test (FCSRT) - delayed free recall ≤ 8
    - FCSRT free recall - learning ≤22
    - Wechsler memory scale revised (WMS-r) story delayed recall (%) ≤75%
    - WMS-r delayed recall of figures (%) ≤ 75%

  Non-memory
    - Trial making test A ≥60
    - Trial making test B ≥150
    - Symbol digit substitution test ≤35 (120 sec)
    - Category fluency ≤16 (60 sec)

  B) Evidence for underlying AD pathology within 1 year prior to screening by either:
    1. CSF β-amyloid (1-42/1-40)*10 ratio <1 and/or total-tau >350 pg/mL and/or phospho-tau >60 pg/mL OR
    2. MRI evidence for medial temporal lobe atrophy (MTA score 1 or higher) OR
    3. Abnormal FDG-PET compatible with AD type changes
- Age 55-85 years
- MMSE ≥24 (participants with very low education (≤6 years) MMSE ≥20 is allowed)
- Written informed consent from participant as well as caregiver
- Availability of a responsible caregiver
- Putative non-prescription/prescription cognitive enhancers (e.g. ginkgo) and statins are not excluded but the dosage should be stable for at least 3 months prior to randomisation. Doses should be kept stable during the study if possible.
- When there is progression to dementia/AD during the trial, participants are allowed to stay in the trial and start registered AD medication, according to clinician's judgment. In addition, participants switch to the use of active product.

Exclusion criteria

- Dementia according to Diagnostic and Statistical Manual of Mental Disorders, Fourth Edition (DSM-IV)
- Use of omega-3 preparations
- Historical use of donepezil, rivastigmine, galantamine and/or memantine
- Alcohol or drug abuse
- A concomitant serious disease
- Major depressive disorders (DSM-IV)
- Intake of vitamin B6, B12, folic acid, vitamin C and/or E >200% Recommended Daily Intake
- Participation in any other clinical trial in the last 30 days
- Participants with an MRI (or CT) scan consistent with a diagnosis of stroke, intracranial bleeding, mass lesion, or normal pressure hydrocephalus. Those participants with a MRI scan demonstrating minimal white matter changes and up to 1-2 lacunar infarcts which are judged to be clinically insignificant are allowed.

Neuropsychological test battery (NTB)

The cognitive test battery of the LipiDiDiet study consisted of a modified version of the NTB[1] and included the following 16 tests:

- Five cognitive subtests from the Consortium to Establish a Registry for Alzheimer's Disease (CERAD)[2]
    - CERAD 10-word list learning immediate recall [score range 0-30]
    - CERAD 10-word list learning delayed recall [score range 0-10]
    - CERAD 10-word list learning recognition [score range 0-20]
    - CERAD constructional praxis copy test [score range 0-11]
    - CERAD constructional praxis recall test [score range 0-11]

- Five cognitive subtests from the WMS-r[3]
  - WMS-r logical verbal memory immediate recall [score range 0-25]
  - WMS-r logical verbal memory delayed recall [score range 0-25]
  - WMS-r digit span total score [score range 0-24]
  - WMS-r visual paired associates immediate recall [score range 0-18]
  - WMS-r visual paired associates immediate delayed recall [score range 0-6]
- Category fluency [correct items in 60 sec][4]
- Boston naming test, 30-item [score range 0-30][5]
- Concept Shifting Test (CST)[6]
  - CST condition A [score range 0-150], corrected for basic motor speed
  - CST condition B [score range 0-150], corrected for basic motor speed
  - CST condition C [score range 0-300], corrected for basic motor speed
- Letter digit substitution test [correct items in 60 sec][7]

MRI acquisition and analyses

For logistic reasons imaging results were more readily available than CSF results, therefore most participants were included based on MRI as evidence for underlying AD pathology before a CSF result became available. MRI acquisitions were performed according to local site protocol and consisted of a 3D T1-weighted anatomical scan for volumetric analysis, 2D FLAIR for visual rating of white matter hyperintensities and a susceptibility weighted image (SWI), or gradient echo for visual rating of microbleeds. Some participants received only a clinical scan at baseline without a 3D T1, or a CT scan. Clinical scans were used for visual rating when possible, but not included in the automated analysis of brain volumes. Acquisition parameters for the 3D T1-weighted scanning protocol differed per site. Kuopio: Siemens Avanto 1.5 T with a magnetisation prepared rapid acquisition gradient echo (MPRAGE) sequence (1.17 mm x 1.17 mm x 1.2 mm, repetition time (TR) = 2400 ms, echo time (TE) 3.5 ms, inversion time (TI) 1000 ms, flip angle = 6 degrees); Amsterdam: Siemens Sonata 1.5 T with a MPRAGE sequence (1.2 mm x 1.2 mm x 1.2 mm, TR = 2400ms, TE 5.06ms, TI 1000ms, FA = 6 degrees) or a GE Signa 1.5 T with a fast spoiler gradient echo (FSPGR) sequence (0.98 mm x 0.98 mm x 1.2 mm, TR = 12.424 ms, TE 5.18ms, TI 450 ms, FA = 12 degrees); Regensburg: Siemens Aera 1.5 T with a MPRAGE sagittal sequence (0.98 mm x 0.98 mm x 1 mm, TR = 2140 ms or 2060 ms, TE 5.97 ms, TI 1120 ms, FA = 15 degrees); Tübingen: Siemens Aera 1.5 T with a MPRAGE sequence (1.2 mm x 1.2 mm x 1.2 mm, TR = 2400 ms, TE 2.29 ms, TI 1000 ms, FA = 6 degrees); Mannheim: Siemens TrioTim 3T with a MPRAGE sequence (1 mm x 1mm x 1 mm, TR = 2300 ms, TE 3.03 ms, TI 900 ms, FA = 9 degrees); Woerden: Philips Achieva 1.5T with a FFE sequence (0.95 mm x 0.95 mm x 1.2 mm, TR = 6.91 ms, TE 3.16 ms, FA = 6 degrees); Stockholm: Siemens Symphony 1.5T with an MPRAGE sequence (1.05 mm x 1.05 mm x 1 mm, TR = 2400 ms, TE 2.6 ms, TI 1000 ms, FA = 6 degrees OR 0.98 mm x 0.98 mm x 1 mm, TR = 2400 ms, TE 2.6 ms, TI 1000 ms, FA = 6 degrees); Homburg: Siemens Skyra 3T with an MPRAGE sequence (1 mm x 1 mm x 1 mm, TR = 2300 ms, TE 2.96 ms, TI 900 ms, FA = 9 degrees); Heidelberg: participants were scanned on different machines without standardised protocol, only those with good quality 3DT1 were included in automated analysis.

All MRI scans were transferred to a central location (VU University Medical Centre, Amsterdam, the Netherlands) for central analysis. MRI scans were acquired at baseline, month 12, and month 24. 289 baseline, 238 12-month and 217 24-month scans were received at VU Medical Centre. 254 baseline, 235 12-month and 207 24-month scans were suitable for automated analysis. Baseline volumetric measures and changes over time were assessed with automated measurements. We assessed change between baseline and month 12 and baseline and month 24. For participants for whom no suitable baseline scan was available, we measured change between month 12 and month 24.

Baseline hippocampal and whole brain volume and changes over time were obtained using Multiple-Atlas Propagation and Segmentation with Hippocampal Boundary Shift Integral (MAPS-HBSI).[8] Ventricular volumes were obtained using the longitudinal processing stream in FreeSurfer 5.3.0,[9] by summing left and right lateral and inferior ventricular volumes. Brain segmentations were visually inspected and excluded in case of poor quality. The volume at follow-up was defined as the baseline volume (or volume at 12-month scan in case of missing baseline) plus the change relative to baseline. Volumes were corrected for head size by multiplying the volumes with the scaling factor obtained from SIENAX analyses at baseline (or 12-month scan in case of missing baseline).[10] 218 baseline, 223 12-month and 201 24-month hippocampal volumes were used for final analyses, including n=18 with scans at months 12 and 24 only. 190 baseline, 194 12-month and 175 24-month for whole brain volume (including n=15 with scans at months 12 and 24 only) and 238 baseline, 222 12-month and 197 24-month for ventricular volume (including n=25 with scans at months 12 and 24 only).

Biochemical analyses

Blood was collected in tubes containing ethylenediaminetetraacetic acid and in serum separator clot activator tubes (for plasma and serum, respectively). All samples were centrifuged immediately (2000 g, 10 min), except for serum which was allowed to clot at room temperature (10 min) first. Plasma and serum aliquots were stored at −80°C until analysis at a central laboratory. The fatty acid composition of the total lipid fraction in plasma was analysed qualitatively on a gas chromatograph after extraction of the lipids from the plasma and a methylation step.[11] Serum HDL cholesterol and LDL cholesterol were analysed enzymatically on the Abbott ARCHITECT platform.

All clinical sites followed the same protocol for CSF sampling to minimise pre-analytical confounders. In short, lumbar puncture was performed in the morning (9-11 AM), and a fixed volume (10 mL) was tapped by gravity drip (no catheter or syringe pull was used). In the case of visible minor CSF tap haemorrhage, the first mL of CSF was discarded, and thereafter CSF was tapped into a new tube. The CSF was immediately sent to the local laboratory, where the sample was centrifuged (2000 g, 10 min, +4°C) to eliminate cells and other insoluble material, aliquoted in polypropylene tubes with a screw cap, and stored at −70-80°C pending analysis. Local analysis results were used for eligibility, CSF cut-offs were adjusted to local lab measures if needed.

The central CSF analyses were performed in the Clinical Neurochemistry Laboratory at Sahlgrenska University hospital, Mölndal, Sweden, which is accredited by the Swedish board of Accreditation and follows strict protocol for quality control, including internal quality control (QC) for approval of each plate, as detailed in Palmqvist et al..[12] All samples were analysed at the same time using the same batch of reagents by board-certified laboratory technicians who were blind to clinical information. CSF Aβ38, Aβ40 and Aβ42 concentrations were measured using the MSD Abeta Triplex (Meso Scale Discovery, Rockville, Maryland) and the Aβ42/40 ratio was calculated as [Aβ42/Aβ40]x10. CSF total tau (T-tau) and tau phosphorylated at threonine181 (P-tau) were measured using INNOTEST sandwich ELISAs (Fujirebio, Ghent, Belgium).

Storage conditions of study product

The investigator was responsible for storing the study product in a secure, limited access storage area. The study product was to be stored at room temperature, protected from extremes of light and humidity.

Relevant protocol amendments: changes to methods and trial outcomes after start of the trial

<u>General</u>

- Seven additional study centres were initiated after the original four centres in order to ensure sufficient recruitment in a reasonable time frame. This resulted in a total of 11 study centres.

<u>Protocol amendments</u>

- Several protocol amendments were submitted at each study centre to include one to four optional 12-month extension studies to the 24-month intervention study.
- An amendment to the protocol was implemented in October 2012 to describe the change in procedures when the active product became commercially available (from September 2012 onwards, varying by country). From that moment onwards, participants progressing to dementia were allowed to switch to the use of active product (either alone or in combination with registered AD medication, according to clinician's judgement) rather than remaining on double-blind study product. This was done without the participant or any member of the study personnel, including the investigators and study-site staff, being unblinded to the originally received double-blind treatment. That is, original treatment codes were not unmasked and the active product was provided as a new product with a different label, but with similar appearance and flavours as the double-blind study products. In addition, confirmation from all study sites was obtained at the end of the study that unmasking envelopes were not opened. All participants were promptly and properly informed and provided separate written informed consent.
- In February 2013, all protocol versions were amended to describe the interim analysis, the installation of an independent data monitoring committee, and the transition in the production of study product (i.e. tetra packs to plastic bottles).
- An amendment to the protocol was implemented in April 2013 to specify the primary endpoint of the study. Advances in AD research suggested that an NTB containing multiple neuropsychological tests may not be sensitive enough to measure intervention effects in similar populations.[13–15] From the NTB total, five tests that were suggested to be most sensitive to intervention effects over time in an early AD/prodromal AD population were selected and shown to be affected by the active product compared with control in previous trials.[16,17] The protocol amendment specified the following NTB composite scores as secondary endpoints: NTB memory domain, NTB executive function domain, and NTB total.
- Based on new insights from literature[18–22] and after careful consideration by the LipiDiDiet trial steering committee of the sensitivity and relevance of specific outcome measures, a protocol

amendment was implemented in June 2015, prior to database lock, to reduce the total number of secondary outcome parameters by re-categorising the secondary and exploratory endpoints.

Definition of the per-protocol population

The per-protocol population consists of all participants from the modified intention-to-treat population, excluding participants or distinct visits of participants with major protocol deviations.

The following rules have been applied for exclusion of *participants* from the per-protocol population:

- Participants who did not comply with the major eligibility criteria (i.e. episodic memory impairment, underlying AD pathology, age, MMSE, exclusion dementia diagnosis) (n=13)
- Participants who did not consume any study product (n=1)
- Participants residing in the same household (potentially risk for mix-up of study product), who after unmasking turned out to have been randomised to different study products (n=2)

The following rules have been applied for exclusion of distinct *visits* of participants from the per-protocol population:

- Use of nutritional supplements comparable to the active product or use of omega-3 preparations (9 visits, n=4)
- Start, stop, or change in dose of relevant medications (i.e. antipsychotics, antidepressants, anxiolytics, anticholinergics, or antiepileptics, opioids, or hypnotics) that were expected to affect the primary endpoint (32 visits, n=28)
- Adverse events or serious adverse events that were expected to affect the primary endpoint (4 visits, n=4)
- No regular study product intake (i.e. low intake, as defined by 15 products or fewer over 8 weeks, before study visits) (36 visits, n=29)
- Overall very low study product intake (≤60%) (5 visits, n=3)
- Missing data about study product intake (24 visits, n=9)
- A gap of ≥14 days in study product intake prior to 24-month (end of study) visit (6 visits, n=6)

Joint model analyses and results

To correct for potential bias (data missing not at random) due to dropout, we performed a sensitivity analysis using a joint model. In this model we combined 1) a mixed model that is comparable to our original primary model (MM) with 2) a Cox proportional hazards model for time to dropout, including the same terms as in the primary model. The two are processed separately and subsequently combined into a single joint model, accounting for associations between study dropout and measurement values.[23] Joint model analyses were performed using the JM package in 'R'.[24]

The joint models led to comparable results for the CDR-SB (estimated mean treatment difference of –0·55, 95% CI –1·04 to –0·06, p=0·027), and MRI total hippocampal volume (0·12, 95% CI 0·02 to 0·22, p=0·015), suggesting that any bias due to dropout did not affect our results.

REFERENCES

1. Harrison J, Minassian SL, Jenkins L, Black RS, Koller M, Grundman M. A neuropsychological test battery for use in Alzheimer disease clinical trials. *Arch Neurol* 2007; **64**: 1323–9.
2. Morris JC, Heyman A, Mohs RC, et al. The Consortium to Establish a Registry for Alzheimer's Disease (CERAD). Part I. Clinical and neuropsychological assessment of Alzheimer's disease. *Neurology* 1989; **39**: 1159–65.
3. Wechsler D. Wechsler Memory Scale-Revised Manual. San Diego: Psychological Corp; 1987.
4. Lezak MD, Howieson DB, Loring DW. Neuropsychological Assessment. New York: Oxford University Press; 2004.
5. Kaplan E, Goodglass H, Weintraub S. The Boston Naming Test. Philadelphia: Lea & Febige; 1983.
6. Van der Elst W, Van Boxtel MP, Van Breukelen GJ, Jolles J. The Concept Shifting Test: adult normative data. *Psychol Assess* 2006; **18**: 424–32.
7. Van der Elst W, van Boxtel MP, van Breukelen GJ, Jolles J. The Letter Digit Substitution Test: normative data for 1,858 healthy participants aged 24-81 from the Maastricht Aging Study (MAAS): influence of age, education, and sex. *J Clin Exp Neuropsychol* 2006; **28**: 998–1009.
8. Leung KK, Barnes J, Ridgway GR, et al. Automated cross-sectional and longitudinal hippocampal volume measurement in mild cognitive impairment and Alzheimer's disease. *Neuroimage* 2010; **51**: 1345–59.
9. Reuter M, Schmansky NJ, Rosas HD, Fischl B. Within-subject template estimation for unbiased longitudinal image analysis. *Neuroimage* 2012; **61**: 1402–18.
10. Smith SM, Zhang Y, Jenkinson M, et al. Accurate, robust, and automated longitudinal and cross-sectional brain change analysis. *Neuroimage* 2002; **17**: 479–89.
11. Morrison WR, Smith LM. Preparation of Fatty Acid Methyl Esters and Dimethylacetals from Lipids with Boron Fluoride--Methanol. *J Lipid Res* 1964; **5**: 600–8.
12. Palmqvist S, Zetterberg H, Blennow K, et al. Accuracy of brain amyloid detection in clinical practice using cerebrospinal fluid beta-amyloid 42: a cross-validation study against amyloid positron emission tomography. *JAMA Neurol* 2014; **71**: 1282–9.
13. Salloway S, Sperling R, Gilman S, et al. A phase 2 multiple ascending dose trial of bapineuzumab in mild to moderate Alzheimer disease. *Neurology* 2009; **73**: 2061–70.
14. Salloway S, Sperling R, Keren R, et al. A phase 2 randomized trial of ELND005, scyllo-inositol, in mild to moderate Alzheimer disease. *Neurology* 2011; **77**: 1253–62.
15. Vellas B, Andrieu S, Sampaio C, Coley N, Wilcock G, European Task Force G. Endpoints for trials in Alzheimer's disease: a European task force consensus. *Lancet Neurol* 2008; **7**: 436–50.
16. Scheltens P, Kamphuis PJ, verhey FRJ, et al. Efficacy of a medical food in mild Alzheimer's disease: A randomized, controlled trial. *Alzheimers Dement* 2010; **6**: 1–10.e1.
17. Scheltens P, Twisk JW, Blesa R, et al. Efficacy of Souvenaid in mild Alzheimer's disease: results from a randomized, controlled trial. *J Alzheimers Dis* 2012; **31**: 225–36.
18. Cano SJ, Posner HB, Moline ML, et al. The ADAS-cog in Alzheimer's disease clinical trials: psychometric evaluation of the sum and its parts. *J Neurol Neurosurg Psychiatry* 2010; **81**: 1363–8.
19. Goldberg TE, Koppel J, Keehlisen L, et al. Performance-based measures of everyday function in mild cognitive impairment. *Am J Psychiatry* 2010; **167**: 845–53.
20. Raghavan N, Samtani MN, Farnum M, et al. The ADAS-Cog revisited: novel composite scales based on ADAS-Cog to improve efficiency in MCI and early AD trials. *Alzheimers Dement* 2013; **9**: S21–31.
21. Cedarbaum JM, Jaros M, Hernandez C, et al. Rationale for use of the Clinical Dementia Rating Sum of Boxes as a primary outcome measure for Alzheimer's disease clinical trials. *Alzheimers Dement* 2013; **9**: S45–55.
22. U.S. Department of Health and Human Services Food and Drug Administration Center for Drug Evaluation and Research (CDER). Guidance for Industry, Alzheimer’s Disease: Developing Drugs for the Treatment of Early Stage Disease, Draft guidance. 2013. http://www.fda.gov/downloads/Drugs/GuidanceComplianceRegulatoryInformation/Guidances/UCM338287.pdf (accessed 20 December 2016).
23. Moll van Charante EP, Richard E, Eurelings LS, et al. Effectiveness of a 6-year multidomain vascular care intervention to prevent dementia (preDIVA): a cluster-randomised controlled trial. *Lancet* 2016; **388**: 797–805.
24. Rizopoulos D. JM: An R Package for the Joint Modelling of Longitudinal and Time-to-Event Data. *J Statistical Software* 2010; **35**: 1–33.

ADDITIONAL TABLES AND FIGURES

Supplementary table 1: Nutritional composition of Fortasyn Connect, the specific nutrient combination in the medical food Souvenaid

**Component**	**Amount per daily dose** (125 mL)
EPA	300 mg
DHA	1200 mg
Phospholipids	106 mg
Choline	400 mg
UMP	625 mg
Vitamin E (alpha-TE)	40 mg
Vitamin C	80 mg
Selenium	60 µg
Vitamin B12	3 µg
Vitamin B6	1 mg
Folic acid	400 µg

EPA=eicosapentaenoic acid. DHA=docosahexaenoic acid.
UMP=uridine monophosphate. TE=tocopherol equivalents.

Supplementary table 2: Parameters used to assess eligibility for prodromal Alzheimer's disease at screening

	**Control** (n=158)	**Active** (n=153)
**Episodic memory disorder**		
Memory*		
FCSRT free recall – learning	13.6 (7.67) [0-30]	14.4 (7.71) [0-34]
FCSRT delayed free recall	4.2 (3.71) [0-15]	4.8 (3.80) [0-14]
WMS-r story delayed recall	4.6 (4.42) [0-23]	5.1 (4.44) [0-17]
WMS-r delayed recall of figures	8.5 (9.15) [0-34]	10.8 (10.25) [0-38]
FCSRT free recall - learning ≤22	134/156 (86%)	128/152 (84%)
FCSRT delayed free recall ≤8	133/155 (86%)	124/152 (82%)
WMS-r story delayed recall (%) ≤75%	109/156 (70%)	103/153 (67%)
WMS-r delayed recall of figures (%) ≤75%	130/143 (91%)	124/144 (86%)
Non-memory*		
Trial making test A	59.5 (30.2) [18-200]	62.7 (34.1) [21-293]
Trial making test B	160.0 (74.9) [48-360]	166.6 (78.5) [51-403]
Symbol digit substitution test	41.1 (15.09) [4-90]	39.2 (16.14) [6-89]
Category fluency	17.5 (5.77) [6-44]	18.2 (5.16) [5-31]
Trial making test A ≥60	59/156 (38%)	63/152 (41%)
Trial making test B ≥150	64/144 (44%)	68/141 (48%)
Symbol digit substitution test ≤35	58/156 (37%)	66/153 (43%)
Category fluency ≤16	70/157 (45%)	53/153 (35%)
**Underlying AD pathology (local)**		
CSF*		
Abnormal amyloid	20/64 (31%)	22/57 (39%)
Total-tau	64/82 (78%)	57/70 (81%)
Phospho-tau	62/81 (77%)	58/70 (83%)
MTA score ≥1*	128/137 (93%)	126/130 (97%)
Abnormal FDG-PET compatible with AD type changes*	14/20 (70%)	11/13 (85%)
**Underlying AD pathology (centrally analysed)**		
CSF*		
Aβ-42 < 450 pg/mL	41/61 (67%)	33/46 (72%)
(Aβ-42/Aβ-40) x 10 < 1	56/61 (92%)	38/46 (83%)
Total-tau >350 pg/mL	53/61 (87%)	39/46 (85%)
Phospho-tau >60 pg/mL	44/61 (72%)	30/46 (65%)
MTA score ≥1*	121/141 (86%)	120/138 (87%)

Data are mean (SD) [min-max] or n/N (%).
*Data not available for all randomised participants. Percentages are calculated based on number of participants with available data.
FCSRT=Free and Cued Selective reminding test. WMS-r=Wechsler Memory Scale Revised. AD=Alzheimer's disease. CSF=cerebrospinal fluid. MTA=medial temporal lobe atrophy. FDG-PET=fluorodeoxyglucose positron emission tomography.

Supplementary table 3: Classification according to the IWG-1, IWG-2, and NIA-AA criteria based on centrally analysed CSF and MRI data

Criteria	Definition	%	n/N
IWG-1 (2007) Prodromal AD	At least one AD biomarker	91%	262/287
IWG-2 (2014) Prodromal AD	Abnormal amyloid AND abnormal tau	76%	81/107
NIA-AA (2011) Low AD likelihood	Normal amyloid and normal neuronal injury markers	0%	0/107
NIA-AA (2011) High AD likelihood	Abnormal amyloid and abnormal neuronal injury markers	87%	93/107
NIA-AA (2011) IAP	Abnormal amyloid and normal neuronal injury markers	1%	1/107
NIA-AA (2011) SNAP	Normal amyloid and abnormal neuronal injury markers	12%	13/107
NIA-AA (2011) Intermediate AD likelihood	One marker tested (=MTA) and abnormal	86%	155/180
NIA-AA (2011) Inconclusive / uninformative group	One marker tested (=MTA) and normal	14%	25/180
Abnormal amyloid	Abnormal amyloid (A+)	88%	94/107

Biomarkers used were as follows: CSF amyloid were β-amyloid 42, β-amyloid 42/40 ratio; CSF tau were total-tau, phospho-tau; neuronal injury markers were CSF total-tau, phospho-tau, MTA.
AD=Alzheimer's Disease. CSF=cerebrospinal fluid. IAP=isolated amyloid pathology. IWG=International Working Group. MTA=medial temporal lobe atrophy. NIA-AA=National Institute of Aging-Alzheimer Association. SNAP=suspected non-Alzheimer pathophysiology.

Supplementary table 4: Distribution of baseline MMSE by treatment group (ITT)

Baseline MMSE	**Control** (n=158)<br>n (%)	**Active** (n=153)<br>n (%)
20	0	1 (0·7%)
21	0	3 (2·0%)
22	0	1 (0·7%)
23	5 (3·2%)	4 (2·6%)
24	19 (12·0%)	23 (15·1%)
25	14 (8·9%)	18 (11·8%)
26	27 (17·1%)	19 (12·5%)
27	25 (15·8%)	32 (21·1%)
28	32 (20·3%)	27 (17·8%)
29	24 (15·2%)	16 (10·5%)
30	12 (7·6%)	8 (5·3%)

MMSE=MMSE=mini-mental state examination.
ITT=intention-to-treat.

Supplementary table 5: Primary endpoint and main secondary endpoints, unadjusted for baseline MMSE

	Difference†	MM[1]	MMs[2]	Effect size[3]
	Estimate (95% CI)	p value	p value	Cohen's *d*
**Primary endpoint**				
**NTB primary (z-score)**				
Modified intention-to-treat	0·067 (−0·071, 0·206)	0·339	0·411	0·12
Per-protocol	0·115 (−0·041, 0·270)	0·147	0·090	0·20
**Secondary endpoints**				
**NTB memory domain (z-score)**				
Modified intention-to-treat	0·111 (−0·054, 0·275)	0·185	0·200	0·15
Per-protocol	0·164 (−0·021, 0·349)	0·082	0·042	0·23
**NTB executive function domain (z-score)**				
Modified intention-to-treat	−0·070 (−0·205, 0·066)	0·311	0·131	-0·13
Per-protocol	−0·016 (−0·160, 0·129)	0·829	0·574	-0·03
**NTB total (z-score)**				
Modified intention-to-treat	0·006 (−0·099, 0·110)	0·917	0·987	0·01
Per-protocol	0·047 (−0·066, 0·159)	0·414	0·456	0·12
**CDR-SB***				
Modified intention-to-treat	−0·54 (−0·96, −0·13)	0·010	0·010	0·31
Per-protocol	−0·66 (−1·10, −0·22)	0·004	0·004	0·40
**MRI total hippocampal volume (cm^3)**				
Modified intention-to-treat	0·12 (0·03, 0·20)	0·006	0·005	0·22
Per-protocol	0·12 (0·03, 0·20)	0·011	0·009	0·19
**MRI whole brain volume (cm^3)**				
Modified intention-to-treat	3·49 (−2·88, 9·86)	0·280	0·294	0·20
Per-protocol	4·97 (−1·99, 11·93)	0·159	0·125	0·29
**MRI ventricular volume (cm^3)***				
Modified intention-to-treat	−1·29 (−2·61, 0·03)	0·056	0·051	0·21
Per-protocol	−1·33 (−2·70, 0·04)	0·057	0·051	0·19

*Higher scores indicate worse performance. For all other endpoints, higher scores indicate better performance.
†Difference is calculated as (active − control) based on least squares means for change from baseline over 24 months as estimated in the mixed model.
[1]MM (mixed model): linear mixed model for longitudinal data with change from baseline as outcome, baseline score and baseline MMSE as covariates, and real measurement time as a continuous variable. P value for effect of intervention over 24 months.
[2]MMs (sensitivity model): mixed model for repeated measures with change from baseline as outcome, baseline score and baseline MMSE as covariates, and planned visit time as a categorical variable. P value for effect of intervention over 24 months.
[3]Cohen's *d* standardised effect size calculated based on the mean treatment difference over 24 months as estimated from the mixed model and the pooled SD. Results are presented so that a positive effect size indicates improved performance in the active vs. control group and vice versa.
MMSE=mini-mental state examination. CI=confidence interval. NTB=neuropsychological test battery. CDR-SB=clinical dementia rating - sum of boxes. MRI=magnetic resonance imaging.

Supplementary table 6: Predefined subgroup analysis for participants with baseline MMSE ≥26

	**Control** (n=120)		**Active** (n=102)		**Difference‡**	**MM**[1]	**MMs**[2]
	Mean (SD)†	n	Mean (SD)†	n	Estimate (95% CI)	p value	p value
**NTB primary endpoint (z-score)**							
Modified intention-to-treat	−0·144 (0·492)	110	−0·034 (0·474)	92	0·075 (−0·083, 0·233)	0·350	0·265
Per-protocol	−0·169 (0·535)	97	0·042 (0·422)	78	0·136 (−0·041, 0·312)	0·131	0·044
**NTB memory domain (z-score)**							
Modified intention-to-treat	−0·178 (0·605)	110	0·011 (0·608)	92	0·131 (−0·062, 0·323)	0·181	0·100
Per-protocol	−0·218 (0·645)	97	0·101 (0·560)	78	0·201 (−0·019, 0·421)	0·073	0·017
**NTB executive function domain (z-score)**							
Modified intention-to-treat	−0·055 (0·444)	110	−0·170 (0·475)	91	−0·100 (−0·251, 0·051)	0·191	0·118
Per-protocol	−0·058 (0·478)	97	−0·094 (0·384)	77	−0·051 (−0·208, 0·106)	0·522	0·494
**NTB total (z-score)**							
Modified intention-to-treat	−0·075 (0·347)	109	−0·067 (0·371)	92	−0·025 (−0·139, 0·090)	0·673	0·732
Per-protocol	−0·088 (0·364)	96	−0·017 (0·327)	78	0·016 (−0·106, 0·139)	0·794	0·600
**CDR-SB***							
Modified intention-to-treat	1·11 (1·79)	95	0·45 (1·33)	79	−0·67 (−1·13, −0·21)	0·005	0·004
Per-protocol	1·14 (1·94)	78	0·30 (1·12)	66	−0·82 (−1·32, −0·33)	0·001	0·001
**MRI total hippocampal volume (cm^3)**							
Modified intention-to-treat	−0·44 (0·34)	81	−0·30 (0·29)	64	0·11 (0·01, 0·21)	0·030	0·030
Per-protocol	−0·43 (0·34)	71	−0·29 (0·30)	56	0·12 (0·02, 0·22)	0·025	0·023
**MRI whole brain volume (cm^3)**							
Modified intention-to-treat	−24·94 (22·39)	69	−18·70 (17·03)	53	5·60 (−1·35, 12·54)	0·113	0·136
Per-protocol	−24·65 (21·35)	60	−17·89 (17·14)	45	6·04 (−1·44, 13·51)	0·112	0·153
**MRI ventricular volume (cm^3)***							
Modified intention-to-treat	7·95 (5·85)	82	5·85 (4·77)	64	−0·96 (−2·56, 0·65)	0·238	0·236
Per-protocol	7·59 (5·03)	72	5·26 (4·53)	56	−1·08 (−2·74, 0·57)	0·195	0·189

*Higher scores indicate worse performance. For all other endpoints, higher scores indicate better performance.
†Data for active and control groups are presented as observed mean change from baseline at month 24 and SD. n is number of participants with at least one post-baseline value in the mixed model.
‡Difference is calculated as (active − control) based on least squares means for change from baseline over 24 months as estimated in the mixed model.
[1]MM (mixed model): linear mixed model for longitudinal data with change from baseline as outcome, baseline score and baseline MMSE as covariates, and real measurement time as a continuous variable. P value for effect of intervention over 24 months.
[2]MMs (sensitivity model): mixed model for repeated measures with change from baseline as outcome, baseline score and baseline MMSE as covariates, and planned visit time as a categorical variable. P value for effect of intervention over 24 months.
MMSE=mini-mental state examination. SD=standard deviation. CI=confidence interval. NTB=neuropsychological test battery. CDR-SB=clinical dementia rating - sum of boxes. MRI=magnetic resonance imaging.

Supplementary table 7: Other secondary endpoints

	**Control** (n=158)		**Active** (n=153)		**Difference†**	**MM[1]**	**MMs[2]**
	Mean (SD)*	n	Mean (SD)*	n	Estimate (95% CI)	p value	p value
**Serum HDL cholesterol (mmol/L)**							
Modified intention-to-treat	−0·011 (0·219)	126	0·057 (0·231)	120	0·070 (0·014, 0·126)	0·016	0·015
Per-protocol	−0·008 (0·234)	105	0·076 (0·239)	103	0·087 (0·023, 0·151)	0·009	0·022
**Serum LDL cholesterol (mmol/L)**							
Modified intention-to-treat	0·041 (0·666)	126	−0·089 (0·621)	120	−0·142 (−0·329, 0·044)	0·134	0·172
Per-protocol	0·061 (0·639)	105	−0·105 (0·666)	103	−0·188 (−0·394, 0·018)	0·074	0·201
**Plasma DHA (%)**							
Modified intention-to-treat	−0·211 (0·637)	143	2·400 (1·029)	138	2·569 (2·379, 2·759)	<0·0001	<0·0001
Per-protocol	−0·252 (0·672)	133	2·380 (0·962)	128	2·603 (2·396, 2·811)	<0·0001	<0·0001
**Plasma EPA (%)**							
Modified intention-to-treat	−0·249 (0·862)	143	1·140 (0·850)	138	1·224 (1·041, 1·406)	<0·0001	<0·0001
Per-protocol	−0·269 (0·931)	133	1·245 (0·759)	128	1·292 (1·086, 1·499)	<0·0001	<0·0001

*Data for active and control groups are presented as observed mean change from baseline at month 24 and SD. n is number of participants with at least one post-baseline value in the mixed model.
†Difference is calculated as (active − control) based on least squares means for change from baseline over 24 months as estimated in the mixed model.
[1]MM (mixed model): linear mixed model for longitudinal data with change from baseline as outcome, baseline score and baseline MMSE as covariates, and real measurement time as a continuous variable. P value for effect of intervention over 24 months.
[2]MMs (sensitivity model): mixed model for repeated measures with change from baseline as outcome, baseline score and baseline MMSE as covariates, and planned visit time as a categorical variable. P value for effect of intervention over 24 months.
SD=standard deviation. CI=confidence interval. HDL=high-density lipoprotein. LDL=low-density lipoprotein. DHA=docosahexaenoic acid. EPA=eicosapentaenoic acid. MMSE=mini-mental state examination.

Supplementary table 8: Progression to dementia (ITT)

	% (n/N)
**Control**	37·3% (59/158)
**Active**	40·5% (62/153)

ITT=intention-to-treat.

Supplementary table 9: Baseline characteristics of the per-protocol population

	**Control** (n=153)*	**Active** (n=142)*
Age (years) [range]	70·8 (6·0) [54-84]	71·6 (6·7) [54-86]
Sex		
Men	70 (46%)	76 (54%)
Women	83 (54%)	66 (47%)
Ethnic origin		
White	152 (99·3%)	141 (99·3%)
Black	0 (0·0%)	1 (0·7%)
Other	1 (0·7%)	0 (0·0%)
Education (years)	10·6 (3·6)	10·5 (3·9)
Mini-Mental State Examination	27·0 (1·9)	26·5 (2·0)
ApoE ε4 genotype†		
Carrier	89/140 (64%)	79/131 (60%)
Non-carrier	51/140 (36%)	52/131 (40%)
Cognitive measures (composite z-score)		
NTB primary endpoint	−0·012 (0·692)	0·039 (0·656)
NTB memory domain	−0·002 (0·816)	0·015 (0·836)
NTB executive function domain	−0·011 (0·726)	0·035 (0·691)
NTB total	−0·029 (0·574)	0·032 (0·549)
CDR-SB	1·72 (1·15)	1·80 (1·12)
MRI brain volumes (cm^3)		
Total hippocampal volume	5·65 (1·21)	5·59 (1·07)
Whole brain volume	1375·02 (83·11)	1369·74 (79·39)
Ventricular volume	53·66 (23·89)	58·05 (26·29)

Data are mean (SD), n (%), or n/N (%).
*Respective visits of participants were additionally excluded in case of major protocol deviations. Data based on participants with at least one follow-up visit in the per-protocol data set.
†Data not available for all randomised participants. Percentages are calculated based on number of participants with available data.
ApoE=apolipoprotein E. NTB=neuropsychological test battery. CDR-SB=clinical dementia rating - sum of boxes. MRI=magnetic resonance imaging. SD=standard deviation.

Supplementary table 10: CDR-SB in subgroups defined by baseline MMSE

Subgroups of MMSE ≥		Control*		Active*		Difference†	MM[1]	MMs[2]
		Mean (SD)	n	Mean (SD)	n	Estimate (95% CI)	p value	p value
**24**	Modified ITT	1·12 (1·72)	117	0·55 (1·35)	106	−0·57 (−0·99, −0·15)	0·008	0·008
	Per-protocol	1·07 (1·82)	96	0·40 (1·15)	89	−0·67 (−1·13, −0·22)	0·004	0·004
**25**	Modified ITT	1·13 (1·76)	104	0·49 (1·34)	91	−0·67 (−1·12, −0·22)	0·004	0·003
	Per-protocol	1·11 (1·89)	86	0·29 (1·07)	75	−0·85 (−1·33, −0·37)	0·0007	0·0006
**26**	Modified ITT	1·11 (1·79)	95	0·45 (1·33)	79	−0·67 (−1·13, −0·21)	0·005	0·004
	Per-protocol	1·14 (1·94)	78	0·30 (1·12)	66	−0·81 (−1·30, −0·32)	0·001	0·001
**27**	Modified ITT	1·08 (1·84)	77	0·55 (1·39)	63	−0·62 (−1·13, −0·11)	0·017	0·017
	Per-protocol	1·06 (1·98)	66	0·31 (1·18)	53	−0·82 (−1·34, −0·29)	0·003	0·003
**28**	Modified ITT	1·24 (2·03)	55	0·37 (1·36)	43	−0·98 (−1·61, −0·36)	0·002	0·002
	Per-protocol	1·36 (2·21)	48	0·02 (0·96)	37	−1·36 (−2·00, −0·71)	<0·0001	<0·0001
**29**	Modified ITT	1·20 (2·16)	29	0·23 (0·90)	21	−1·05 (−2·05, −0·05)	0·040	0·034
	Per-protocol	1·39 (2·40)	24	0·00 (0·80)	19	−1·38 (−2·44, −0·32)	0·013	0·010

Baseline MMSE was found to be a possible effect modifier for CDR-SB based on interaction term in the mixed model (per-protocol p=0·053).
*Data for active and control groups are presented as observed mean change from baseline at month 24 and SD. n is number of participants with at least one post-baseline value in the mixed model. Higher scores indicate worse performance.
†Difference is calculated as (active − control) based on least squares means for change from baseline over 24 months estimated in the mixed model.
[1]MM (mixed model): linear mixed model for longitudinal data with change from baseline as outcome, baseline score and baseline MMSE as covariates, and real measurement time as a continuous variable. P value for effect of intervention over 24 months.
[2]MMs (sensitivity model): mixed model for repeated measures with change from baseline as outcome, baseline score and baseline MMSE as covariates, and planned visit time as a categorical variable. P value for effect of intervention over 24 months.
CDR-SB=clinical dementia rating - sum of boxes. MMSE=mini-mental state examination. SD=standard deviation. CI=confidence interval. ITT=intention-to-treat.

Supplementary table 11: Observed means (SD) by visit for primary endpoint and main secondary endpoints (mITT)

	Baseline<br>Mean (SD)	Month 6<br>Mean (SD)	Month 12<br>Mean (SD)	Month 24<br>Mean (SD)
**Primary endpoint**				
**NTB primary endpoint (z-score)**				
Control	−0·000 (0·679)	0·019 (0·699)	0·012 (0·754)	0·072 (0·791)
Active	−0·002 (0·701)	0·037 (0·762)	0·042 (0·778)	0·225 (0·691)
**Secondary endpoints**				
**NTB memory domain (z-score)**				
Control	0·025 (0·817)	0·081 (0·808)	0·030 (0·933)	0·080 (0·939)
Active	−0·018 (0·872)	0·030 (0·951)	0·020 (0·967)	0·293 (0·899)
**NTB executive function domain (z-score)**				
Control	−0·011 (0·714)	0·015 (0·769)	0·008 (0·792)	0·083 (0·787)
Active	0·006 (0·710)	−0·021 (0·750)	0·015 (0·775)	0·037 (0·749)
**NTB total (z-score)**				
Control	−0·017 (0·565)	0·005 (0·572)	0·028 (0·612)	0·049 (0·688)
Active	0·015 (0·572)	0·018 (0·616)	0·067 (0·645)	0·166 (0·615)
**CDR-SB***				
Control	1·75 (1·14)	-	2·29 (1·74)	2·69 (2·08)
Active	1·87 (1·17)	-	2·15 (1·60)	1·99 (1·61)
**MRI total hippocampal volume (cm^3)**				
Control	5·70 (1·25)	-	5·43 (1·24)	5·42 (1·33)
Active	5·62 (1·10)	-	5·38 (1·14)	5·64 (1·14)
**MRI whole brain volume (cm^3)**				
Control	1377·30 (84·08)	-	1362·66 (85·75)	1375·29 (85·17)
Active	1370·56 (81·64)	-	1360·49 (87·51)	1379·13 (90·95)
**MRI ventricular volume (cm^3)***				
Control	53·95 (25·31)	-	59·83 (26·61)	60·91 (27·29)
Active	58·35 (26·66)	-	63·48 (28·95)	56·93 (26·64)

*Higher scores indicate worse performance. For all other endpoints, higher scores indicate better performance.
- not applicable.
CDR-SB=clinical dementia rating - sum of boxes. mITT=modified intention-to-treat. MRI=magnetic resonance imaging. NTB=neuropsychological test battery. SD=standard deviation.

Supplementary table 12: Listing of adverse events contributing to study discontinuation

Participant	Adverse event	Serious adverse event	Relationship to study product
Control	Sudden death*	Yes	Not related
Control	Acute myocardial infarction	Yes	Not related
Control	Diarrhoea	No	Related
Control	Hypersensitivity	No	Related
Control	Urticaria	No	Related
Control	Lactose intolerance and weight increased	No	Related
Active	Psychotic disorder	Yes	Not related
Active	Cerebral haemorrhage	Yes	Not related
Active	Bronchial carcinoma*	Yes	Not related
Active	Infection*†	Yes	Not related
Active	Respiratory failure *†	Yes	Not related
Active	Respiratory failure*‡	Yes	Not related
Active	Eczema	No	Related
Active	Eczema	No	Related
Active	Abdominal pain upper	No	Related
Active	Regurgitation	No	Related
Active	Lactose intolerance	No	Related

Adverse events are presented by MedDRA preferred term.
*Participant died.
†Primary reason for discontinuation as indicated on the Case Report Form was 'Other' rather than 'Adverse Event'. Specification of other: 'death'/ 'participant passed away'.
‡Participant discontinued the study while on active product after progression to dementia. Participant received active product during the double-blind treatment.
For 15 participants the occurrence of adverse events was the primary reason for discontinuation (n=6 in control vs. n=9 in active; p=0·437; figure 1), whereas for two additional participants the occurrence of adverse events contributed to discontinuation, but this was not considered as the primary reason (Supplementary table 10). So in total, for 17 participants the occurrence of adverse events contributed to discontinuation (n=6 in control vs. n=11 in active; p=0·219), of whom for 8 participants the adverse event was classified as a serious adverse event (n=2 in control vs. n=6 in active; p=0·168). Five causes of death were observed due to four different causes. All serious adverse events that contributed to discontinuation were considered as not related to the study product and include the five deaths.

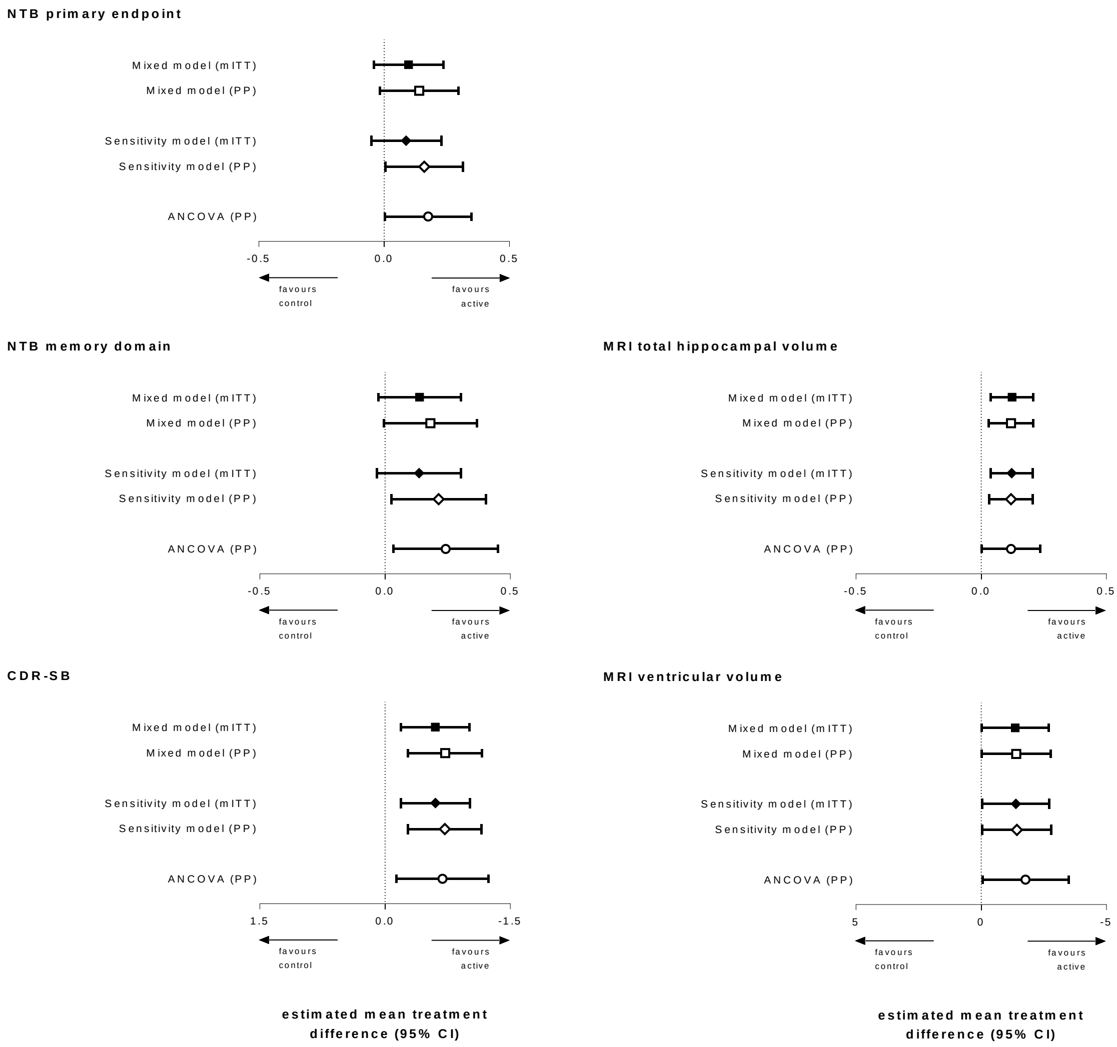

Supplementary figure 1: Forest plots

Data are estimated mean treatment difference and 95% CI for change from baseline over 24 months as estimated by the different statistical models.

NTB=neuropsychological test battery. CDR-SB=clinical dementia rating - sum of boxes. MRI=magnetic resonance imaging. mITT=modified intention-to-treat. PP=per-protocol. CI=confidence interval.
